# A Quantitative Assessment of Upper Limb Motor Function Across Disease Stages in Hereditary Transthyretin Amyloidosis

**DOI:** 10.1111/jns.70127

**Published:** 2026-06-05

**Authors:** Mehrnaz Hamedani, Valeria Prada, Sara Massucco, Edoardo Roveta, Marina Grandis, Chiara Gemelli, Irene Schiavetti, Alessio Signori, Cristina Schenone, Marco Luigetti, Valeria Guglielmino, Maria Ausilia Sciarrone, Francesca Vitali, Luca Guglielmo Pradotto, Fiore Manganelli, Stefano Tozza, Giovanni Palumbo, Anna Mazzeo, Luca Gentile, Massimo Russo, Marcella De Luca, Federica D'Arma, Chiara Pisciotta, Silvia Fenu, Davide Pareyson, Angelo Schenone

**Affiliations:** ^1^ Department of Neuroscience, Rehabilitation, Ophthalmology, Genetics, Maternal and Child Health (DINOGMI) University of Genova Genova Italy; ^2^ Italian Multiple Sclerosis Foundation, AISM Genova Italy; ^3^ Fondazione “DAVID CHIOSSONE” Genova Italy; ^4^ IRCCS Azienda Ospedaliera Metropolitana (AOM) Neurological Clinic Genova Italy; ^5^ Department of Health Sciences (DISSAL) University of Genova Genova Italy; ^6^ IRCCS Azienda Ospedaliera Metropolitana (AOM) Genova Italy; ^7^ Fondazione Policlinico A. Gemelli IRCCS, UOC Neurologia Rome Italy; ^8^ Dipartimento di Neuroscienze Università Cattolica del Sacro Cuore Rome Italy; ^9^ Ivrea Civil Hospital – ASLTO4 Ivrea Italy; ^10^ Department of Neurosciences, Reproductive and Odontostomatology Sciences University of Naples “Federico II” Naples Italy; ^11^ Department of Clinical and Experimental Medicine University of Messina Messina Italy; ^12^ Department of Clinical Neurosciences Fondazione IRCCS Istituto Neurologico Carlo Besta Milan Italy

**Keywords:** amyloidosis, early detection, motor performance, neuropathy, sensitivity, sensor, upper limb

## Abstract

**Background and Aims:**

Hereditary transthyretin amyloidosis (ATTRv) is a multisystemic disease where early neuropathy signs are challenging to detect conventionally. This study aimed to evaluate hand motor performance in ATTRv using the Hand Test System (HTS) across disease stages and examine correlations with standard measures.

**Methods:**

A total of 113 individuals were enrolled: 74 patients with ATTRv (divided according to Familial Amyloid Polyneuropathy [FAP] stage into FAP0: pre‐symptomatic with confirmed *TTR* mutations, *n* = 16; FAP1: mild sensory/motor symptoms, *n* = 40; FAP2: ambulatory with assistance, *n* = 18) and 39 healthy controls. All participants underwent HTS evaluation; 9‐Hole Peg Test (9HPT); handgrip and tripod pinch strength testing; Thumb Opposition Test (TOT); Neuropathy Impairment Score (NIS); Disabilities of the Arm, Shoulder and Hand questionnaire (DASH); and Quality of Life Diabetic Neuropathy (Norfolk QoL‐DN) questionnaire. Group differences were analyzed with analysis of covariance and correlations with Pearson's coefficients.

**Results:**

HTS parameters (Touch Duration, Inter‐Tapping Interval, Movement Rate) significantly differed across stages between the FAP2 and all other groups, with some parameters distinguishing the FAP1 from the control and FAP0 groups. 9HPT impairment was observed only in the FAP2 group. Grip strength showed subtle changes, especially in the right hands of the FAP1 group, while tripod pinch strength declined during advanced stages. Several HTS parameters were correlated with NIS, DASH, Norfolk QoL‐DN, and hand strength. Subclinical carpal tunnel syndrome possibly influenced early‐stage results.

**Interpretation:**

HTS detected stage‐related motor differences in ATTRv and correlated them with standard measures, offering increased sensitivity to early neuropathic changes.

## 1 Introduction

Hereditary transthyretin amyloidosis (ATTRv, v for variant) is an autosomal‐dominant multisystem disorder caused by mutations in the *TTR* gene, which causes the misfolding and extracellular deposition of transthyretin as insoluble amyloid fibrils, predominantly affecting the peripheral nerves, heart, and autonomic structures [1, 2, 3]. Once considered a fatal and incurable disease, ATTRv is now treatable after the introduction of transthyretin stabilizers (e.g., tafamidis, diflunisal, and acoramidis), gene silencers (e.g., patisiran, vutrisiran, inotersen, and eplontersen), and, more recently, investigational gene‐editing therapies [4, 5, 6, 7]. These therapeutic advances emphasize the need for timely diagnosis and sensitive biomarkers capable of detecting disease onset before irreversible damage occurs [8, 9]. More than 120 transthyretin point mutations have been identified, and they are often associated with distinct clinical patterns [10]. While some variants predominantly cause neuropathy and other cardiomyopathies, many exhibit a mixed phenotype with overlapping neurological and cardiac involvement [11, 12]. However, their early motor and sensory changes can be subtle and difficult to detect using standard clinical tools. Although the Neuropathy Impairment Score (NIS) and grip strength assessments are widely used in both clinical and research settings [13, 14], they lack sufficient sensitivity in the earliest stages, where compensatory mechanisms often mask clinical signs [15, 16]. Additionally, the high prevalence of carpal tunnel syndrome (CTS) in patients with ATTRv can confound the interpretation of upper limb dysfunction and complicate the assessment of disease‐specific motor impairment [17, 18]. To address these challenges, quantitative sensor‐based technologies that provide objective, reproducible, and fine‐grained data on motor functions have been increasingly considered [19]. The Hand Test System (HTS) is a validated, rapid, and noninvasive approach that assesses upper limb function across multiple parameters, including Touch Duration (TD), Inter‐Tapping Interval (ITI), and Movement Rate (MR) [20, 21]. In hereditary neuropathies, such as Charcot–Marie–Tooth disease, HTS has demonstrated strong correlations with clinical severity, grip strength, and clinical scales, proving its sensitivity in detecting subtle motor deficits [22]. However, given its prior application in other hereditary and acquired neuropathies, HTS‐derived alterations are likely to reflect non‐specific motor dysfunction rather than disease‐specific signatures. Despite these promising applications, HTS has not yet been applied for ATTRv diagnosis, and its ability to detect early‐stage disease or capture phenomena, such as overwork weaknesses, remains unknown. Furthermore, the relationship between HTS‐derived metrics and traditional clinical measures of ATTRv remains underexplored.

The present study aimed to investigate hand motor performance in patients with ATTRv using HTS and compare results across disease stages. Correlations between HTS parameters and grip strength (handgrip and tripod pinch) as well as clinical scales were also explored, focusing particularly on early‐stage detection and functional asymmetry. We hypothesized that HTS could provide a quantitative assessment of motor impairment across disease stages, with potential utility not only in detecting subtle functional changes but also in tracking disease severity and progression over time.

## 2 Materials and Methods

### 2.1 Study Population

A total of 113 participants were recruited for the study, including 74 patients with a pathogenic mutation in the *TTR* gene and 39 healthy controls. The participants were enrolled to assess their motor performance and quality of life metrics in a clinical setting. The ATTRv patients were further stratified into subgroups based on the clinical stage of Familial Amyloid Polyneuropathy (FAP). A score of 1 (FAP1) indicated mild sensory and/or motor symptoms in the lower limbs without significant impairment in daily activities (ambulatory without assistance); 2 (FAP2) reflected more advanced symptoms with difficulty walking, requiring support (ambulatory with assistance); and 3 (FAP3) represented severe disability, with patients being confined to a wheelchair or bedridden (non‐ambulatory) [23].

In this study, a group of pre‐symptomatic carriers (individuals with genetically confirmed pathogenic variants in the TTR gene but no clinical signs or symptoms of neuropathy) with normal nerve conduction results was included. Although this category is not formally part of the original staging system, it is referred to here as stage FAP0 for clarity and consistency of reporting and analysis. Patients with FAP3 were excluded because of concomitant advanced hand disabilities. The study protocol was approved by the Ethics Committees of the coordinating center (N. Registro CER Liguria: 728/2021—DB id 11 480) and all participating centers. All participants provided written informed consent. The study was conducted in accordance with the Declaration of Helsinki.

### 2.2 Inclusion and Exclusion Criteria

The study enrolled adult participants (aged ≥ 18 years) with a positive genetic analysis of the *TTR* gene, regardless of the disease stage. Both symptomatic and pre‐symptomatic individuals were eligible for inclusion. A control group of healthy individuals was also recruited, comprising participants without a history of peripheral neuropathy or any comorbid conditions known to affect hand function (e.g., carpal tunnel syndrome).

Participants were excluded if they were receiving medications known to affect motor performance, such as neuroleptics or centrally acting muscle relaxants, or if they reported uncontrolled hand pain (visual analog scale score > 5). Additional exclusion criteria included a history of hand surgery resulting in significant mobility impairment or comorbidities that could interfere with upper limb motor assessment (e.g., brain stroke, Parkinson's disease, advanced rheumatoid arthritis, severe osteoarthritis, or post‐traumatic hand deformities). Carpal tunnel release surgery was not considered as an exclusion criterion unless it was associated with substantial functional limitations. For the ATTRv group, participants were excluded if genetic confirmation of the diagnosis was lacking or if they were more than 10 years younger than the predicted age of disease onset (PADO) [9].

### 2.3 Evaluation Protocol

Each participant underwent a standardized assessment using the HTS, which included a sensorized glove with five conductive sensors embedded on the palmar surface of each distal phalanx. These sensors detected contact during thumb‐to‐finger opposition movements. The evaluation comprised two 30‐s tasks performed with the eyes closed: (1) Finger Tapping (FT), which involved tapping the thumb to the index finger as rapidly as possible and (2) the index–middle–ring–little (IMRL) sequence, which required sequential thumb opposition to the index, middle, ring, and little fingers. Before starting, the operator provided standardized instructions and informed the participants that the test would begin by following the three acoustic signals. The participants were instructed to perform thumb opposition movements at their maximum speed for the entire duration of each task, while keeping their eyes closed. All assessments were conducted in a quiet environment to minimize external distractions and ensure optimal concentration. The key parameters recorded during these tasks were: (1) Touch Duration (TD), which is the contact time between the thumb and another finger, measured in milliseconds (ms); (2) Inter Tapping Interval (ITI), which is the time between the end of contact between the thumb and one finger and the beginning of the next contact, also measured in ms; and (3) Movement Rate (MR), which is the frequency of the motor task, measured in hertz (Hz), calculated as 1/(TD + ITI).

In addition to the HTS evaluation, the participants completed a battery of motor and functional assessments. These included the 9‐Hole Peg Test (9HPT) [24], which measures fine motor coordination and dexterity; grip and tripod pinch strength testing using dynamometry (Citec CT 3001, CIT Technics BV, Groningen, The Netherlands) [25]; the Thumb Opposition Test (TOT), evaluating thumb mobility and opposition function [26]; the Norfolk Quality of Life‐Diabetic Neuropathy (Norfolk QoL‐DN) questionnaire [27], which evaluates quality of life related to neuropathy symptoms and their impact on daily activities; the NIS [28], which quantifies the severity of peripheral neuropathy based on muscle strength, reflexes, and sensory deficits; and the Disability of the Arm, Shoulder, and Hand (DASH) questionnaire [29], assessing upper limb function and symptoms.

### 2.4 Statistical Analysis

Descriptive data were expressed as means with standard deviations for continuous variables or as absolute and relative frequencies for categorical variables. Baseline differences among groups were assessed using the Kruskal–Wallis U test for continuous variables due to the non‐parametric distribution of the data and the chi‐squared test for associations between categorical variables. Group differences in continuous outcomes were examined using an analysis of covariance (ANCOVA) adjusted for age and sex based on baseline differences. Pairwise comparisons were conducted with Bonferroni correction, and the results are reported as mean differences (adjusted for age and sex), along with standard errors and *p*‐values. *p*‐values less than 0.05 were rounded to three decimal places; those equal to or greater than 0.05 were rounded to two decimal places. Correlations between all parameters were assessed using Pearson's correlation coefficient and visualized in a correlation matrix generated using the corrplot package in R, employing a color gradient ranging from red (negative correlations) to yellow (weak correlations), then green (positive correlations).

## 3 Results

### 3.1 Demographic and Clinical Characteristics of the Patients

The study population comprised 74 patients with ATTRv and 39 healthy controls. All participants successfully completed the full clinical and functional assessment protocols. Table 1 summarizes the patients' demographic and clinical characteristics. Among the participants with ATTRv, 66.2% were men (*n* = 49) and 33.8% were women (*n* = 25), and the mean age was 64.8 ± 11.73 years. Of these, 16 were classified as having FAP0. The control group was similarly balanced by age, with a mean of 63 ± 13.58 years; 46.2% were men (*n* = 18) and 53.8% were women (*n* = 21). Within the ATTRv cohort, the patients were classified into three clinical stages according to disease severity. The FAP0 included 16 participants, accounting for 21.6% of the ATTRv sample, with a mean age of 50 ± 7.1 years and a predominance of women (62.5%). The FAP1 group, representing symptomatic individuals, comprised 40 patients (54.1%), mostly men (67.5%), with a mean age of 66 ± 10.06 years. The FAP2 group included 18 patients (24.3%), predominantly men (88.9%), with a mean age of 72 ± 6.64 years. When considering all four groups (FAP0, FAP1, FAP2, and controls), significant differences were found by sex (*p* < 0.001) and age (*p* = 0.004). Neurophysiological assessment in the FAP0 subgroup (*n* = 16) identified mild abnormalities indicative of CTS in five patients. In three cases, motor fibers were also involved, with right‐sided findings in two patients and left‐sided in one.

**TABLE 1:** Demographic and outcome values.

	Parameter values by group			
	Healthy, *N* = 39	FAP0, *N* = 16	FAP1, *N* = 40	FAP2, *N* = 18
Gender				
Females	21 (53.8%)	10 (62.5%)	13 (32.5%)	2 (11.1%)
Males	18 (46.2%)	6 (37.5%)	27 (67.5%)	16 (88.9%)
	*Mean ± SD*	*Mean ± SD*	*Mean ± SD*	*Mean ± SD*
Age (year)	63 ± 13.58	50 ± 7.10	66 ± 10.06	72 ± 6.64
FT TD right (ms)	120.8 ± 26.80	114.2 ± 28.44	155.8 ± 71.31	296.3 ± 138.15
FT TD left (ms)	139.0 ± 28.81	125.4 ± 37.28	161.5 ± 62.20	319.8 ± 138.30
FT ITI right (ms)	91.3 ± 27.65	89.4 ± 22.38	117.8 ± 41.41	406.4 ± 401.10
FT ITI left (ms)	98.3 ± 29.38	91.5 ± 20.58	118.1 ± 35.18	317.8 ± 379.90
FT MR right (Hz)	4.8 ± 0.68	5.1 ± 0.85	4.0 ± 1.09	2.0 ± 1.11
FT MR left (Hz)	4.3 ± 0.72	4.7 ± 0.78	3.8 ± 0.96	2.0 ± 0.85
IMRL TD right (ms)	200.4 ± 48.46	208.3 ± 73.03	286.5 ± 109.79	461.0 ± 154.79
IMRL TD left (ms)	218.8 ± 47.86	218.2 ± 63.09	294.6 ± 99.18	529.4 ± 173.68
IMRL ITI right (ms)	147.3 ± 53.76	127.0 ± 31.03	191.5 ± 69.06	332.0 ± 219.44
IMRL ITI left (ms)	139.4 ± 48.27	128.9 ± 32.99	202.9 ± 114.68	274.0 ± 142.56
IMRL MR right (Hz)	3.0 ± 0.64	3.1 ± 0.77	2.3 ± 0.67	1.3 ± 0.59
IMRL MR left (Hz)	2.9 ± 0.58	3.0 ± 0.77	2.3 ± 0.74	1.4 ± 0.51
9HPT right (s)	19.5 ± 2.91	19.5 ± 2.75	39.7 ± 52.73	108.1 ± 79.88
9HPT left (s)	20.5 ± 4.52	20.7 ± 3.45	35.0 ± 26.66	72.8 ± 45.36
Handgrip right (N)	188.5 ± 72.56	196.9 ± 79.34	136.3 ± 75.72	64.2 ± 62.72
Hand grip left (N)	183.4 ± 67.90	193.0 ± 79.46	144.1 ± 74.99	71.1 ± 61.69
Tripod pinch right (N)	89.6 ± 31.18	95.9 ± 33.54	74.2 ± 38.77	29.4 ± 24.88
Tripod pinch left (N)	82.9 ± 29.00	89.7 ± 27.92	72.8 ± 37.09	33.6 ± 22.48
TOT right (score)	9.7 ± 0.79	9.4 ± 0.73	7.4 ± 1.65	5.2 ± 1.93
TOT left (score)	9.7 ± 0.68	9.8 ± 0.58	7.6 ± 1.65	5.8 ± 2.49
Norfolk QOL‐DN (score)	—	1.8 ± 6.23	36.9 ± 28.07	76.2 ± 22.72
NIS TOT (score)	—	0.3 ± 0.68	30.5 ± 25.22	88.9 ± 28.94
NIS AAII (score)	—	0.3 ± 0.68	18.6 ± 16.96	50.0 ± 14.79
DASH (score)	—	2.9 ± 6.70	33.1 ± 29.03	72.8 ± 13.21

Abbreviations: 9HPT: Nine Hole Peg Test; AAII: lower limbs; DASH: Disabilities of the Arm, Shoulder and Hand; FAP0: familial amyloid polyneuropathy stage 0; FAP1: familial amyloid polyneuropathy stage 1; FAP2: familial amyloid polyneuropathy stage 2; FT: finger tapping; Hz: hertz; IMRL: Index–Middle–Ring–Little sequence; ITI: Inter‐Tapping Interval; MR: Movement Rate; ms: milliseconds; N: Newton; NIS TOT: Neuropathy Impairment Score Total; QOL‐DN: Quality of Life‐Diabetic Neuropathy; s: seconds; SD: standard deviation; TD: Touch Duration; TOT: Thumb Opposition Test.

**FIGURE 1:**
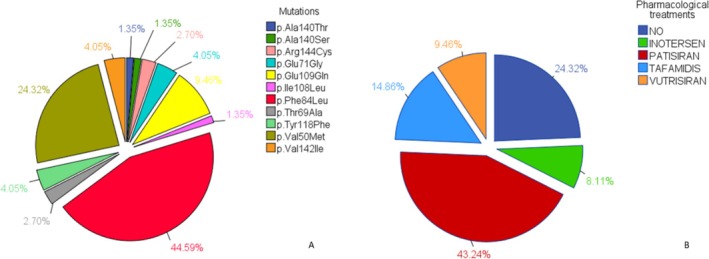
Pie chart of ATTRv mutations (A) and pharmacological treatments (B).

Genetic testing has revealed diverse mutational profiles. As shown in Figure 1A, the p.Phe84Leu variant was the most prevalent, detected in 44.59% of the patients (*n* = 33). The p.Val50Met mutation was identified in 24.32% of the patients (*n* = 18), whereas the p.Glu109Gln mutation was identified in 9.46% (*n* = 7). Other less common variants included p. Tyr118Phe and p.Glu71Gly (both at 4.05%, 3 patients each) as well as p.Val142Ile and p.Arg144Cys (4.05% and 2.7%, respectively), while several rarer forms were found in one patient (1.35%). As illustrated in Figure 1B, 75.7% of the patients (*n* = 56) underwent disease‐modifying therapy. Data were collected between 2022 and 2024, when patients were prescribed patisiran the most (43.24%, 32 patients), followed by tafamidis (14.86%, 11 patients), vutrisiran (9.46%, 7 patients), and inotersens (8.11%, 6 patients). A subset of the patients also received experimental treatments within the framework of pharmacological clinical trials. Notably, 24.3% of the cohort (18 patients) did not receive active treatment at the time of assessment. This subgroup included 16 asymptomatic carriers (FAP0), for whom treatment initiation was not yet indicated, and two patients who had recently transitioned to symptomatic disease (FAP1) and started therapy shortly thereafter.

### 3.2 Hand Test System Tasks

HTS assessments demonstrated significant intergroup differences in the Finger Tapping task performance. As all participants were right‐handed, the results were reported in terms of the dominant (right) and non‐dominant (left) hands. For the right hand, both TD and ITI differed between the FAP2 and FAP1 groups (TD, *p* < 0.001; ITI, *p* = 0.002), FAP0 (TD, *p* < 0.001; ITI, *p* = 0.007), and healthy controls (TD, *p* < 0.001; ITI, *p* = 0.001). For the left hand, TD was significantly prolonged in the FAP2 group compared with the FAP1 (*p* < 0.001), FAP0 (*p* < 0.001), and healthy control (*p* < 0.001) groups, whereas ITI did not differ between disease stages (Tables 1 and 2a).

**TABLE 2a:** Comparative analysis of four groups, *p*‐value and mean differences of Hand Test System (HTS) finger tapping evaluation.

	Hand Test System finger tapping		
	Mean diff.	Std. error	*p*
TD right (ms)			
FAP0‐FAP1	−36.229	24.598	0.86
FAP0‐FAP2	−192.159^a^	34.996	< 0.001
FAP0‐healthy	2.861	23.689	0.99
FAP1‐FAP2	−155.931^a^	29.442	< 0.001
FAP1‐healthy	39.090	16.708	0.13
FAP2‐healthy	195.020^a^	29.346	< 0.001
TD left (ms)			
FAP0‐FAP1	−30.301	23.770	0.99
FAP0‐FAP2	−185.893^a^	33.810	< 0.001
FAP0‐healthy	−6.405	22.888	0.99
FAP1‐FAP2	−155.593^a^	28.504	< 0.001
FAP1‐healthy	23.896	16.134	0.85
FAP2‐healthy	179.488^a^	28.402	< 0.001
ITI right (ms)			
FAP0‐FAP1	−18.028	56.630	0.99
FAP0‐FAP2	−268.258^a^	80.568	0.007
FAP0‐healthy	7.600	54.537	1.000
FAP1‐FAP2	−250.230^a^	67.782	0.002
FAP1‐healthy	25.628	38.466	0.99
FAP2‐healthy	275.858^a^	67.560	0.001
ITI left (ms)			
FAP0‐FAP1	−23.813	52.097	0.99
FAP0‐FAP2	−166.233	74.102	0.16
FAP0‐healthy	−4.083	50.163	0.99
FAP1‐FAP2	−142.419	62.472	0.15
FAP1‐healthy	19.731	35.360	1.000
FAP2‐healthy	162.150	62.248	0.06
MR right (Hz)			
FAP0‐FAP1	1.058^a^	0.308	0.005
FAP0‐FAP2	3.115^a^	0.438	< 0.001
FAP0‐healthy	0.161	0.297	0.99
FAP1‐FAP2	2.056^a^	0.368	< 0.001
FAP1‐healthy	−0.897^a^	0.210	< 0.001
FAP2‐healthy	−2.954^a^	0.368	< 0.001
MR left (Hz)			
FAP0‐FAP1	0.861^a^	0.270	0.011
FAP0‐FAP2	2.598^a^	0.384	< 0.001
FAP0‐healthy	0.293	0.260	0.99
FAP1‐FAP2	1.737^a^	0.324	< 0.001
FAP1‐healthy	−0.569^a^	0.183	0.015
FAP2‐healthy	−2.305^a^	0.323	< 0.001

*Note:* Based on estimated marginal means.

Abbreviations: FAP0: familial amyloid polyneuropathy stage 0; FAP1: familial amyloid polyneuropathy stage 1; FAP2: familial amyloid polyneuropathy stage 2; Hz: hertz; ITI: Inter‐Tapping Interval; MR: Movement Rate; ms: milliseconds; *p*: *p*‐value; SD: standard deviation; TD: Touch Duration.

^a^ The mean difference is significant at the 0.05 level.

Right‐hand MR results differed significantly between the groups, except for the comparison between the healthy control and FAP0 groups. Specifically, patients in the FAP2 group had a lower right‐hand MR than those in the FAP1 (*p* < 0.001), FAP0 (*p* < 0.001), and healthy control groups (*p* < 0.001). The FAP1 group also showed lower right‐hand MR than the FAP0 group (*p* = 0.005) and healthy controls (*p* < 0.001). Similar results were observed for the left hand, where the MR differed significantly across groups, except between the FAP0 and healthy control groups (FAP2 vs. FAP1: *p* < 0.001; FAP2 vs. FAP0: *p* < 0.001; FAP2 vs. healthy controls: *p* < 0.001; FAP1 vs. FAP0: *p* = 0.011; FAP1 vs. healthy controls: *p* = 0.015) (Tables 1 and 2a).

In the complete sequence task, the TD parameters differed significantly across groups, except for between the FAP0 and healthy control groups (*p* = 0.99 for both hands) and the FAP1 and FAP0 groups (right hand: *p* = 0.37; left hand: *p* = 0.34). Significant differences were found between the FAP2 and FAP1 groups (*p* < 0.001 for both hands), FAP2 and FAP0 groups (*p* < 0.001 for both hands), and FAP2 and healthy control groups (*p* < 0.001 for both hands). Additionally, expression in the FAP1 group differed from that in the healthy controls (right hand, *p* = 0.002; left hand, *p* < 0.001; Tables 1 and 2b).

**TABLE 2b:** Comparative analysis of four groups, *p*‐value and mean differences of Hand Test System (HTS) complete sequences evaluation.

	Hand Test System complete sequences		
	Mean diff.	Std. error	*p*
TD right (ms)			
FAP0‐FAP1	−57.631	30.518	0.37
FAP0‐FAP2	−216.015^a^	42.737	< 0.001
FAP0‐healthy	28.351	29.123	0.99
FAP1‐FAP2	−158.384^a^	34.884	< 0.001
FAP1‐healthy	85.982^a^	20.809	< 0.001
FAP2‐healthy	244.366^a^	34.754	< 0.001
TD left (ms)			
FAP0‐FAP1	−55.358	28.720	0.34
FAP0‐FAP2	−341.732^a^	42.130	< 0.001
FAP0‐healthy	18.683	27.413	0.99
FAP1‐FAP2	−286.374^a^	36.054	< 0.001
FAP1‐healthy	74.042^a^	19.523	0.002
FAP2‐healthy	360.416^a^	35.855	< 0.001
ITI right (ms)			
FAP0‐FAP1	−46.076	28.434	0.65
FAP0‐FAP2	−177.762^a^	39.817	< 0.001
FAP0‐healthy	−3.447	27.134	0.99
FAP1‐FAP2	−131.686^a^	32.502	0.001
FAP1‐healthy	42.629	19.387	0.18
FAP2‐healthy	174.315^a^	32.380	< 0.001
ITI left (ms)			
FAP0‐FAP1	−48.315	29.123	0.60
FAP0‐FAP2	−174.307^a^	42.722	0.001
FAP0‐healthy	7.296	27.798	0.99
FAP1‐FAP2	−125.992^a^	36.560	0.005
FAP1‐healthy	55.612^a^	19.797	0.036
FAP2‐healthy	181.603^a^	36.358	< 0.001
MR right (Hz)			
FAP0‐FAP1	0.719^a^	0.220	0.009
FAP0‐FAP2	1.588^a^	0.309	< 0.001
FAP0‐healthy	0.005	0.210	0.99
FAP1‐FAP2	0.869^a^	0.252	0.005
FAP1‐healthy	−0.714^a^	0.150	< 0.001
FAP2‐healthy	−1.583^a^	0.251	< 0.001
MR left (Hz)			
FAP0‐FAP1	0.614^a^	0.224	0.044
FAP0‐FAP2	1.588^a^	0.328	0.000
FAP0‐healthy	−0.026	0.214	1.000
FAP1‐FAP2	0.974^a^	0.281	0.005
FAP1‐healthy	−0.640^a^	0.152	0.000
FAP2‐healthy	−1.614^a^	0.279	0.000

*Note:* Based on estimated marginal means.

Abbreviations: FAP0: familial amyloid polyneuropathy stage 0; FAP1: familial amyloid polyneuropathy stage 1; FAP2: familial amyloid polyneuropathy stage 2; HZ: herz; ITI: Inter‐Tapping Interval; MR: Movement Rate; ms: milliseconds; *p*: *p*‐value; SD: standard deviation; TD: Touch Duration.

^a^ The mean difference is significant at the 0.05 level.

FAP2 patients had significantly different right ITI compared with those with FAP1 (*p* = 0.001), FAP0 (*p* < 0.001), and healthy controls (*p* < 0.001). Similar differences were observed for the left hand (FAP2 vs. FAP1, *p* = 0.005; FAP2 vs. healthy controls, *p* < 0.001), and the FAP1 group also differed from the healthy control group (*p* = 0.036; Tables 1 and 2b).

Finally, the MR results showed significant group differences, except between the FAP0 and healthy control groups. For the right hand, the FAP2 group had a lower MR than the FAP1 (*p* = 0.005), FAP0 (*p* < 0.001), and healthy control groups (*p* < 0.001), whereas the FAP1 group exhibited a lower MR than both the FAP0 (*p* = 0.009) and healthy control groups (*p* < 0.001). Similar results were observed for the left hand (FAP2 vs. FAP1, *p* = 0.005; FAP2 vs. FAP0, *p* < 0.0001; FAP2 vs. healthy controls, *p* < 0.0001; FAP1 vs. FAP0, *p* = 0.044; FAP1 vs. healthy controls, *p* < 0.0001) (Tables 1 and 2b).

### 3.3 Manual Dexterity and Hand Strength Evaluation

The 9HPT demonstrated significant performance differences in patients of the FAP2 group compared with those in the FAP1, FAP0, and healthy control groups for both the right and left hands (*p* < 0.001 for all comparisons).

Handgrip strength, assessed using a dynamometer, was also significantly lower in the FAP2 group than in the FAP1 (*p* < 0.001), FAP0 (*p* < 0.001), and healthy control (*p* = 0.001) groups. On the right hand, patients in the FAP1 group showed weaker grip strength than the healthy controls (*p* < 0.0001). For the left hand, grip strength in the FAP2 group was significantly lower than that in FAP1 (*p* = 0.024), FAP0 (*p* < 0.001), and healthy control groups (*p* < 0.001); patients in the FAP1 group also differed significantly from the healthy controls (*p* = 0.004). Notably, right‐hand grip strength was significantly lower in the FAP1 group than that in the healthy controls, showing a trend toward significance against FAP0 (*p* = 0.08).

The Tripod Pinch Test showed significant results, with the FAP2 group differing significantly from the FAP1 (right: *p* < 0.001; left: *p* = 0.004), FAP0 (*p* < 0.001 for both hands), and healthy control groups (*p* < 0.001 for both sides) (Tables 1 and 3).

**TABLE 3:** Comparative analysis of four groups, *p*‐value and mean differences of hand functioning evaluation.

	Mean diff.	Std. error	*p*
9HPT right (s)			
FAP0‐FAP1	−10.956	14.710	0.99
FAP0‐FAP2	−88.606^a^	21.137	< 0.001
FAP0‐healthy	5.196	14.164	0.99
FAP1‐FAP2	−77.650^a^	17.938	< 0.001
FAP1‐healthy	16.152	9.982	0.65
FAP2‐healthy	93.802^a^	17.870	< 0.001
9HPT left (s)			
FAP0‐FAP1	−10.859	7.758	0.99
FAP0‐FAP2	−53.736^a^	11.230	< 0.001
FAP0‐healthy	1.145	7.469	0.99
FAP1‐FAP2	−42.877^a^	9.523	< 0.001
FAP1‐healthy	12.004	5.263	0.15
FAP2‐healthy	54.881^a^	9.492	< 0.001
Hand grip right (N)			
FAP0‐FAP1	53.166	21.253	0.08
FAP0‐FAP2	132.111^a^	30.236	< 0.001
FAP0‐healthy	−6.909	20.467	0.99
FAP1‐FAP2	78.945^a^	25.438	0.015
FAP1‐healthy	−60.075^a^	14.436	< 0.001
FAP2‐healthy	−139.020^a^	25.354	< 0.001
Hand grip left (N)			
FAP0‐FAP1	48.062	20.486	0.13
FAP0‐FAP2	120.338^a^	29.145	< 0.001
FAP0‐healthy	−0.777	19.728	0.99
FAP1‐FAP2	72.277^a^	24.520	0.024
FAP1‐healthy	−48.839^a^	13.915	0.004
FAP2‐healthy	−121.115^a^	24.439	< 0.001
Tripod pinch right (N)			
FAP0‐FAP1	15.373	9.856	0.731
FAP0‐FAP2	64.631^a^	14.023	< 0.001
FAP0‐healthy	−1.437	9.492	0.99
FAP1‐FAP2	49.258^a^	11.798	< 0.001
FAP1‐healthy	−16.810	6.695	0.08
FAP2‐healthy	−66.068^a^	11.759	< 0.001
Tripod pinch left (N)			
FAP0‐FAP1	14.449	9.376	0.76
FAP0‐FAP2	55.436^a^	13.340	< 0.001
FAP0‐healthy	2.678	9.030	0.99
FAP1‐FAP2	40.987^a^	11.223	0.002
FAP1‐healthy	−11.771	6.369	0.40
FAP2‐healthy	−52.758^a^	11.186	< 0.001
TOT right (score)			
FAP0‐FAP1	1.626^a^	0.459	0.004
FAP0‐FAP2	4.539^a^	0.653	< 0.001
FAP0‐healthy	−0.595	0.442	0.99
FAP1‐FAP2	2.912	0.549	< 0.001
FAP1‐healthy	−2.221	0.312	< 0.001
FAP2‐healthy	−5.134	0.548	< 0.001
TOT left (score)			
FAP0‐FAP1	1.918^a^	0.501	0.001
FAP0‐FAP2	2.895^a^	0.712	0.001
FAP0‐healthy	−0.200	0.482	0.99
FAP1‐FAP2	0.977	0.599	0.64
FAP1‐healthy	−2.118^a^	0.340	< 0.001
FAP2‐healthy	−3.095^a^	0.597	< 0.001

*Note:* Based on estimated marginal means.

Abbreviations: 9HPT: Nine Hole Peg Test; Diff: difference; FAP0: familial amyloid polyneuropathy stage 0; FAP1: familial amyloid polyneuropathy stage 1; FAP2: familial amyloid polyneuropathy stage 2; N: Newton; *p*: *p*‐value; s: seconds; Std: standard; TOT: Thumb Opposition Test.

^a^ The mean difference is significant at the 0.05 level.

The TOT indicated significant impairment in the FAP2 group. On the right hand, patients in the FAP2 group differed significantly from those in the FAP1 (*p* < 0.001), FAP0 (*p* < 0.001), and healthy control groups (*p* < 0.001). For the left hand, the differences were significant between the FAP2 and FAP0 groups (*p* < 0.001) and the healthy controls (*p* < 0.001), but not between the FAP2 and FAP1 groups (*p* = 0.64). Additionally, TOT scores were lower in the FAP1 group than in the FAP0 (right: *p* < 0.004; left: *p* < 0.001) and healthy control (*p* < 0.001 for both sides) groups (Tables 1 and 3).

### 3.4 Clinical Scales and Questionnaires

The Norfolk QoL‐DN questionnaire, applicable only to patients with ATTRv, showed significant differences across disease stages, with patients in the FAP2 group having higher scores than those in the FAP1 (*p* < 0.001) and FAP0 groups (*p* < 0.001), with FAP1 differing from FAP0 (*p* = 0.023).

Patients in the FAP2 group exhibited significantly higher NIS values than those in the FAP1 (*p* < 0.001) and FAP0 (*p* < 0.001) groups, while those in the FAP1 group had higher NIS scores than those in the FAP0 group (*p* = 0.011). When analyzing the lower‐limb NIS (NIS‐LL) sub‐scores, significant differences were observed between the FAP2 and FAP1 (*p* < 0.001), FAP2 and FAP0 (*p* < 0.001), and FAP1 and FAP0 (*p* = 0.008) groups (Tables 1 and 4).

**TABLE 4:** Comparative analysis of three groups, *p*‐value and mean differences of quality of life and neuropathy evaluation.

	Mean diff.	Std. error	*p*
Norfolk QOL‐DN (score)			
FAP0‐FAP1	−25.039^a^	9.094	0.023
FAP0‐FAP2	−70.981^a^	12.498	< 0.001
FAP1‐FAP2	−45.942^a^	9.671	< 0.001
TOT NIS (score)			
FAP0‐FAP1	−28.048^a^	9.285	0.011
FAP0‐FAP2	−85.958^a^	12.761	< 0.001
FAP1‐FAP2	−57.910^a^	9.874	< 0.001
NIS‐LL (score)			
FAP0‐FAP1	−17.840^a^	5.695	0.008
FAP0‐FAP2	−48.318^a^	7.828	< 0.001
FAP1‐FAP2	−30.479^a^	6.057	< 0.001
DASH (score)			
FAP0‐FAP1	−18.780	8.632	0.10
FAP0‐FAP2	−59.914^a^	11.864	< 0.001
FAP1‐FAP2	−41.133^a^	9.180	< 0.001

*Note:* Based on estimated marginal means.

Abbreviations: DASH: Disability of the Arm, Shoulder and Hand; Diff: difference; NIS TOT: Total Neuropathy Impairment Score; NIS‐LL: Neuropathy Impairment Score in the Lower Limbs; Norfolk QOL‐DN: Norfolk Quality of Life; *p*: *p*‐value; Std: standard.

^a^ The mean difference is significant at the 0.05 level.

The DASH questionnaire, a self‐reported measure of upper limb function, revealed significantly higher disability in patients with FAP2 than in those with FAP1 (*p* < 0.001) and FAP0 (*p* < 0.001). However, no significant differences were found between the FAP1 and FAP0 groups (*p* = 0.10) (Tables 1 and 4).

**FIGURE 2:**
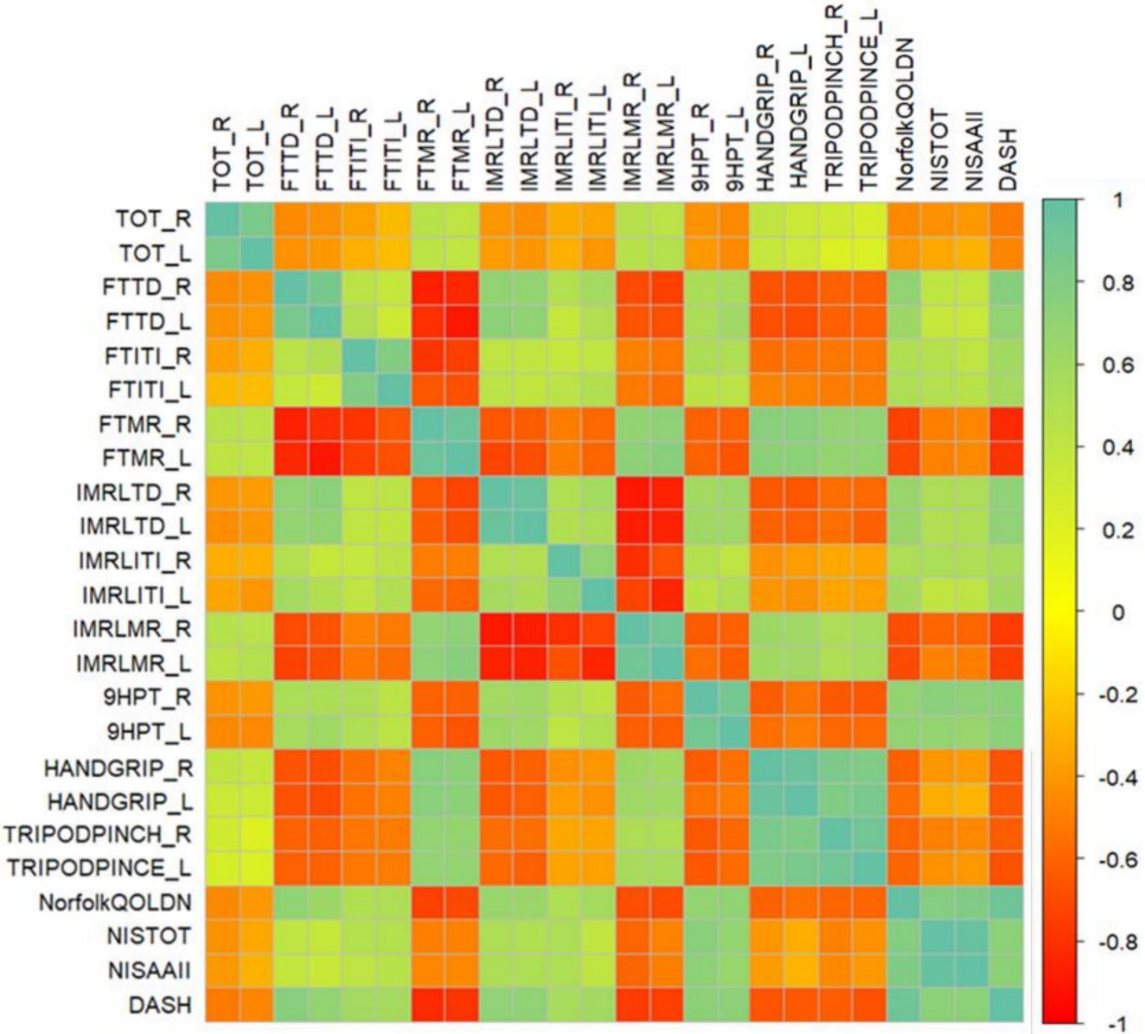
Correlation between clinical scale scores, dynamometry test results, and HTS parameters.

Figure 2 illustrates the consistency of the associations between the HTS‐derived parameters and conventional clinical and functional measures across both hands. TD and ITI were positively correlated with the NIS, DASH, and Norfolk QoL‐DN scores, indicating that longer contact and transition times were linked to greater clinical impairment, higher perceived disability, and poorer quality of life. Conversely, MR was positively associated with grip and tripod pinch strength and negatively correlated with NIS, DASH, and Norfolk scores, reflecting improved motor function and overall clinical condition. Notably, TD and ITI also showed positive correlations with lower‐limb NIS subscores, suggesting that reduced upper‐limb motor performance may reflect more widespread peripheral nervous system involvement. These associations were consistent across both hands. TD and ITI also showed positive correlations with the lower‐limb NIS subscores.

## 4 Discussion

ATTRv is a progressive disease that can be managed with disease‐modifying therapies, which are most effective when initiated early, making timely recognition of clinical signs and symptoms essential [12]. Although numerous studies have explored the cardiac phenotypes of the disease [30], evidence is limited regarding functional biomarkers that reflect neurological involvement, particularly in the early or pre‐symptomatic stages. HTS is a rapid, simple, and noninvasive test that can be completed in approximately 20 min and has proven useful in various neurological disorders, including neuromuscular diseases [31]. However, to date, only a few studies have applied personalized or instrument‐based assessments, such as HTS, for ATTRv [13]. Clinical scales alone are often insufficient to detect early abnormalities, and standard neurophysiological studies offer limited sensitivity in early‐stage disease, as they predominantly evaluate large‐fiber function [32, 33]. Therefore, this study explored the utility of HTS as a complementary tool for assessing hand motor function across different ATTRv disease stages.

While our initial aim included the identification of early motor alterations, the present findings more consistently support the role of HTS as a quantitative marker of disease severity across ATTRv stages. The progressive changes observed in MR, TD, and ITI across FAP stages highlight its potential applicability in monitoring disease progression and functional decline.

HTS parameters, particularly those derived from Finger Tapping and Complete Sequence tasks, revealed functional alterations that were not consistently captured by conventional tests. For instance, in the Finger Tapping task, both TD and ITI were significantly increased in the FAP2 group compared with the other groups. While ITI did not differentiate between FAP2 and FAP1 in the left hand, TD remained significantly elevated, indicating a greater sensitivity to subtle motor slowing and coordination deficits. Meanwhile, MR, another key HTS parameter, showed a progressive decline across disease stages.

The Finger Tapping test is straightforward and can often be performed even in advanced ATTRv stages owing to its compensatory movements. While these compensations may partially mask the extent of the underlying motor impairment, they allow for the continued acquisition of personalized, quantifiable parameters. This is particularly relevant in advanced disease stages, as motor nerve conduction studies may become unfeasible owing to severe axonal degeneration, limiting the use of standard neurophysiological assessments. In contrast, Finger Tapping provides consistent, individualized data over time, making HTS suitable for longitudinal monitoring. In the Complete Sequence task, TD emerged as a discriminative marker, with significant differences observed between the FAP2 and all other groups, as well as between the FAP1 and healthy control groups, but not between the FAP0 and FAP1 groups. This pattern suggests the possibility of subtle changes in FAP0; however, these findings should be interpreted with caution. The absence of statistically significant differences between FAP0 and FAP1, together with the limited sample size of these subgroups, reduces the robustness of conclusions regarding early‐stage or pre‐symptomatic impairment. In addition, the presence of potential confounders such as subclinical CTS and the relatively small size of the FAP0 subgroup further limit the interpretability of these early‐stage differences. Although 5 out of 16 patients presented with CTS, only 3 showed motor alterations (two right‐sided and one left‐sided), and the absence of significant TD differences between FAP0 and FAP1 in either hand makes it unlikely that CTS alone could explain the findings. Instead, the observed changes may reflect early ATTRv‐related motor or sensory involvement. While ATTRv is traditionally characterized by length‐dependent neuropathy predominantly affecting the lower limbs, clinical onset in the upper limbs is relatively frequent in non‐endemic regions, such as Italy [34, 35]. This epidemiological pattern may help explain the early subtle alterations detected by the HTS parameters. ITI values in the Complete Sequence test mirrored these trends, showing significant differences between the FAP2 and all other groups and between the FAP1 and healthy control groups, particularly for the left hand. Collectively, these results indicate that HTS parameters are sensitive to gradations of motor impairment across disease stages, with potential utility in disease staging and monitoring progression. Although subtle changes may be present in early stages, the current data more robustly support the role of HTS as a marker of disease severity rather than as a standalone tool for early detection.

The 9HPT identified significant impairments only in the FAP2 group, confirming its limited sensitivity in detecting early motor or sensory dysfunction, aligning with previous findings that suggest its utility primarily in advanced stages [36]. The handgrip strength measured using dynamometry revealed more nuanced distinctions. In both hands, significant reductions were found in the FAP2 group compared with all other groups. In the FAP1 group, right‐hand grip strength was significantly lower than that in the healthy controls, showing a trend toward significance against FAP0. For the left hand, the FAP1 group showed a significant reduction compared to healthy controls, with no significant differences or trends compared with FAP0. Although motor impairment in the ATTRv may present with asymmetry, the random selection of our sample suggests the need to consider alternative explanations. The observed predominance of impairment in the dominant hand may suggest a role for use‐dependent mechanisms; however, this interpretation remains speculative. Alternative explanations, including sampling variability or subclinical asymmetries in disease expression, should also be considered. Further studies specifically designed to address this aspect are warranted.

The tripod pinch test followed a similar pattern, with significant differences being evident only in the FAP2 group compared to all other groups. These results suggest that while grip strength may detect intermediate impairment, tripod pinch strength may be preserved longer, potentially owing to compensatory strategies or the selective involvement of different muscle groups. TOT showed a clear stage‐dependent decline, with the most pronounced abnormalities observed in the FAP2 group. No significant differences emerged between the FAP2 and FAP1 groups in the left hand; however, patients with FAP1 performed worse than FAP0 patients and controls. This suggests that early‐stage patients may not exhibit detectable impairments, either directly or indirectly, through assessments that rely on the TOT scale.

The Norfolk QoL‐DN QoL scores declined with disease progression, with significant differences between the FAP1 and FAP0 groups, suggesting that subjective perception mirrors objective motor deficits.

The DASH questionnaire also revealed an increase in perceived disability with disease progression. However, the differences between the FAP1 and FAP0 groups were not statistically significant, potentially reflecting compensatory mechanisms in early‐stage disease or the limited sensitivity of patient‐reported outcomes in detecting subtle functional impairments.

The NIS confirmed clear distinctions across all clinical stages, particularly in the lower limb sub‐score, reflecting ATTRv systemic neuropathy beyond the upper limbs assessed by HTS.

Several HTS parameters showed strong correlations with established clinical assessments, such as the Hand Grip Test, total and upper‐limb NIS, Norfolk QoL‐DN questionnaire, and 9HPT. Increased TD and ITI values were correlated with higher NIS scores, reflecting greater impairment, whereas higher MR was associated with better grip and pinch strength. This supports the utility of HTS in providing a comprehensive evaluation of hand motor function through a single standardized assessment. Notably, despite HTS focusing only on upper limb performance, TD and ITI values also correlated with LL‐NIS scores, suggesting that the slowing of upper limb motor function may reflect a greater dysfunction of the peripheral nervous system. The bilateral consistency of these associations further strengthens the interpretation that HTS parameters are sensitive markers of systemic neuropathic involvement in ATTRv.

This study had some limitations that should be considered. We were unable to perform analyses based on specific ATTRv mutations that might influence motor involvement, owing to the limited number of cases in each genetic subgroup. The observed asymmetry in grip strength, particularly on the dominant side, may somehow reflect overuse rather than disease‐specific patterns. However, further research is required for confirmation. In addition, the cross‐sectional design did not allow us to evaluate the evolution of motor function over time, which is essential for assessing HTS in longitudinal follow‐ups. Finally, even if this was considered during interpreting the results, a confounding effect related to the presence of subclinical CTS in some pre‐symptomatic individuals cannot be excluded. Furthermore, the relatively small size of certain subgroups, particularly FAP0 and FAP2, may have limited statistical power and reduced the robustness of between‐group comparisons, especially in early disease stages.

## 5 Conclusion

The present findings supported the integration of quantitative and personalized evaluations into the functional assessment of ATTRv. Although approaches such as the HTS are not intended to replace conventional tools, their sensitivity to changes across disease stages, including detectability in advanced stages, suggests that they can provide complementary information. This may be especially valuable in presymptomatic individuals or when standard assessments yield inconclusive results. In clinical practice, HTS may represent a practical and reproducible tool to complement standard evaluations, particularly in quantifying motor impairment and supporting disease staging. Its role in early detection remains to be further clarified. Future longitudinal studies should explore whether HTS metrics correlate with therapeutic response and clarify whether stage‐1 dominant‐hand grip asymmetry reflects use‐dependent mechanisms.

## Funding

Research grant funding was provided by the Ionis Pharmaceuticals Inc., AstraZeneca Pharmaceuticals, and the research was supported by European Union (101156434).

## Conflicts of Interest

The authors declare no conflicts of interest.

## Data Availability

The datasets generated and analyzed during the current study are not publicly available due to privacy and ethical constraints but are available from the corresponding author upon reasonable request and subject to approval by the institutional review board.
